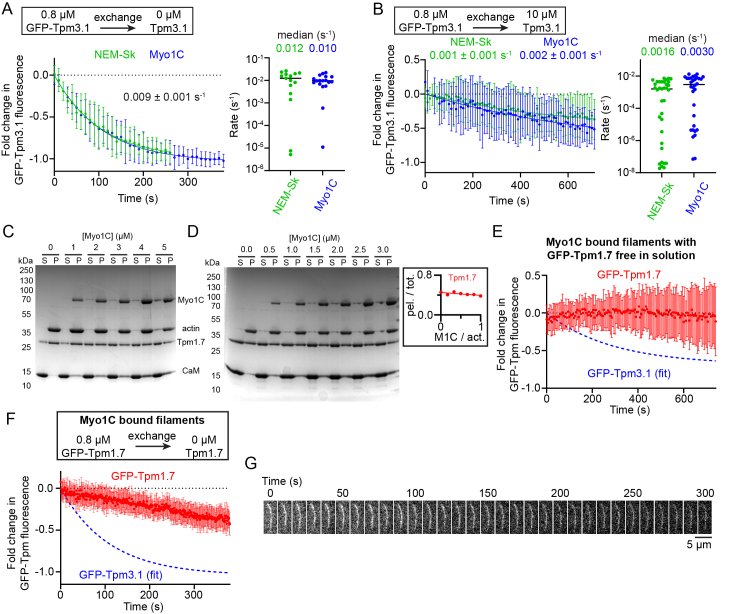# Myosin-1C differentially displaces tropomyosin isoforms altering their inhibition of motility

**DOI:** 10.1016/j.jbc.2024.107539

**Published:** 2024-07-04

**Authors:** Luther W. Pollard, Malgorzata Boczkowska, Roberto Dominguez, E. Michael Ostap

**Affiliations:** Department of Physiology and Pennsylvania Muscle Institute, Perelman School of Medicine, University of Pennsylvania, Philadelphia, Pennsylvania, USA

**Keywords:** myosin-I, nonmuscle tropomyosins, isoform sorting, actin cytoskeleton, myosin regulation, cytoplasmic organization, tropomyosin displacement

## Abstract

Force generation and motility by actomyosin in nonmuscle cells are spatially regulated by ∼40 tropomyosin (Tpm) isoforms. The means by which Tpms are targeted to specific cellular regions and the mechanisms that result in differential activity of myosin paralogs are unknown. We show that Tpm3.1 and Tpm1.7 inhibit Myosin-IC (Myo1C), with Tpm1.7 more effectively reducing the number of gliding filaments than Tpm3.1. Strikingly, cosedimentation and fluorescence microscopy assays revealed that Tpm3.1 is displaced from actin by Myo1C and not by myosin-II. In contrast, Tpm1.7 is only weakly displaced by Myo1C. Unlike other characterized myosins, Myo1C motility is inhibited by Tpm when the Tpm-actin filament is activated by myosin-II. These results point to a mechanism for the exclusion of myosin-I paralogs from cellular Tpm-decorated actin filaments that are activated by other myosins. Additionally, our results suggest a potential mechanism for myosin-induced Tpm sorting in cells.

A fundamental question in cell biology is how does the cell create and spatially regulate distinct architectures of the actin cytoskeleton using a common pool of building blocks? In animal cells, ∼40 tropomyosin (Tpm) isoforms are coiled-coil proteins that assemble along the long-pitch helix of actin filaments to create distinct microfilament compartments (1). Tpms restrict and promote the binding of a variety of proteins that control the dynamics of actin polymerization as well as the assembly of filaments into higher-order structures. The myosin superfamily of actin-based motors represents a major target for regulation by Tpm isoforms, the mechanisms of which are an open area of investigation (2, 3, 4, 5, 6, 7, 8, 9, 10). For example, several Tpm isoforms are known to inhibit class-I myosins while enhancing class-II myosin motility (2, 3, 5, 7, 9, 11, 12, 13, 14, 15). How actin filaments are differentially organized in a Tpm isoform-dependent manner becomes a particularly important question in light of the role of Tpm isoform switching in driving cancer transformation and poor prognosis (16, 17, 18, 19, 20, 21).

Tpm1.7 and Tpm3.1 are the products of the *TPM1* and *TPM3* genes, with Tpm1.7 spanning seven actin subunits and Tpm3.1 spanning six (22). Tpm1.7 is slow to dissociate from actin and has a substantially lower cytosolic fraction than Tpm3.1, which together suggests that Tpm1.7 has a higher affinity for actin in the cell (7, 23). Further, Tpm1.7 is rapidly degraded in the cytosol when not bound to actin, while Tpm3.1 is not (23). These isoforms localize to overlapping and nonoverlapping actin filament populations, especially in stress fibers where they differentially enhance nonmuscle myosin-II activity (1, 7, 9, 23, 24, 25). Notably, the altered ratio of expression of the two Tpms is linked to various cancers (26). In sum, Tpm1.7 and Tpm3.1 have distinct biochemical and physiological properties with clear clinical relevance.

The molecular mechanisms that drive Tpm isoform specificity and selectivity for different myosin classes remain unestablished. However, we can consider Tpm in striated muscle where it adopts distinct positions on actin that regulate myosin activity (27, 28, 29). In the absence of calcium, troponin holds Tpm in a “blocked” position where it sterically prevents the myosin from strong binding. Upon calcium binding to troponin, Tpm enters a “closed” state that inhibits activity but is permissive to weak myosin binding. Myosin binding activates the thin filament by shifting Tpm into an “open” conformation, cooperatively revealing the myosin-binding site (30). Troponin is absent in nonmuscle cells, suggesting a difference in the Tpm regulation of myosin. Notably, nonmuscle Tpms appear to adopt a closed-like position in the absence of myosin binding (31). The diversity of Tpms and myosins outside the context of sarcomeres results in a range of activation, inhibitory, and localization mechanisms. Since the myosin-I paralog Myo1C is regulated by Tpm1.7 and Tpm3.1 in cells and both inhibit Myo1C’s motility *in vitro* (5, 9, 32), we set out to further resolve the mechanism of Tpm-mediated inhibition and the differences between these Tpm isoforms in regulating the mechanochemistry of Myo1C.

## Results

**Figure 1:**
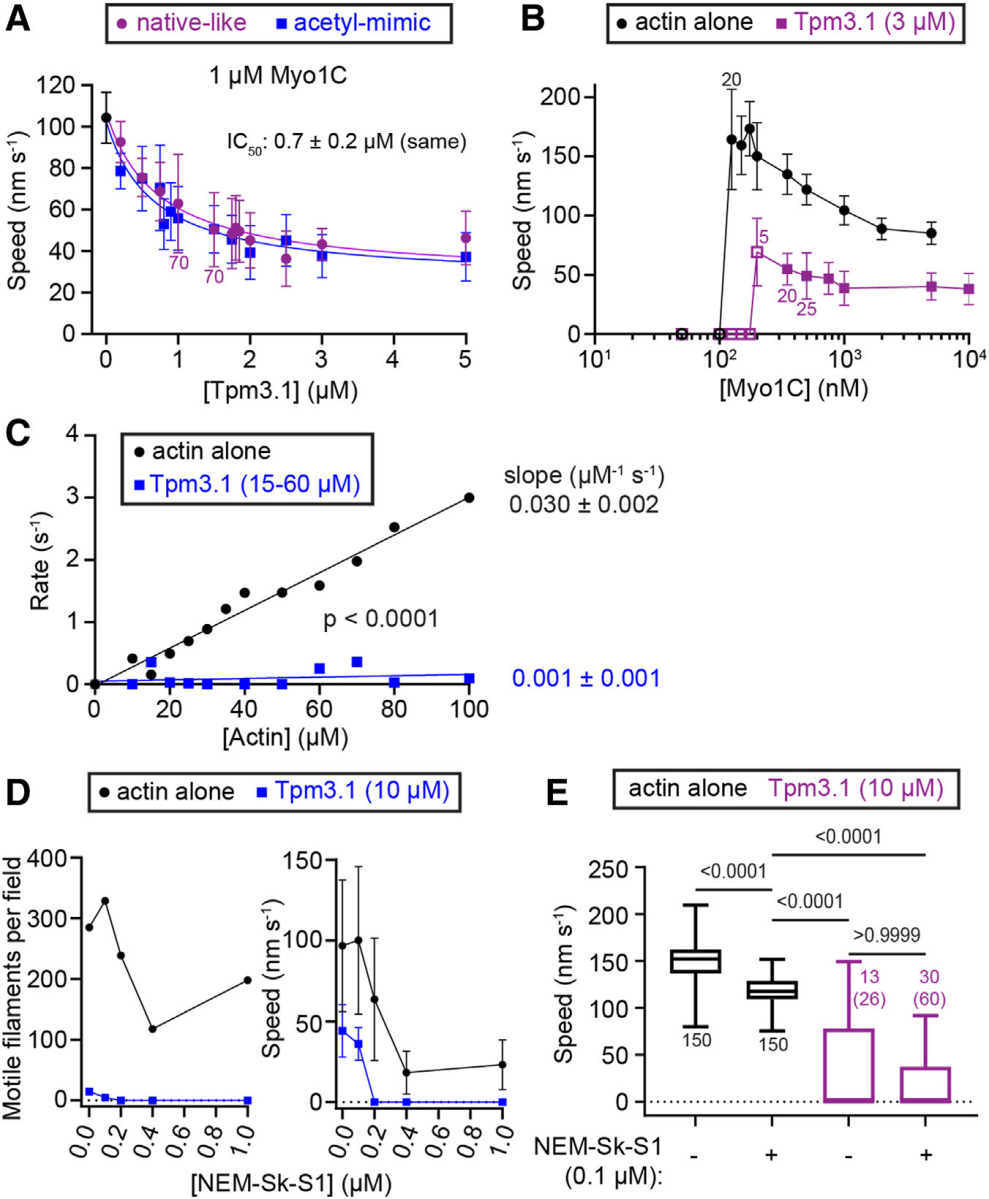
**Tpm3.1 inhibition of Myo1C is not limited by motor availability.***A*, Myo1C actin gliding motility with increasing concentrations of free native- or MAS (acetyl-mimic)- Tpm3.1. Fits, dose-response inhibition model. *B*, effect of (native-) Tpm3.1 on gliding motility with variable concentrations of surface-attached Myo1C. *Solid* lines, connect the points. *A* and *B*, points, mean and SD from individual trials, n = 50 filaments (unless 0 or indicated next to the point). Open symbols, n ≤5 events. *C*, steady-state ATPases. (MAS-) Tpm3.1 and actin alone conditions were performed in parallel. Slopes of linear fits give second-order rates. Data are pooled from three trials employing N = 3 preparations of Myo1C (one experiment per point). *D*, effect of NEM-Sk-S1 (skeletal myosin) on 150 nM Myo1C gliding motility in the absence or presence of (MAS-) Tpm3.1. *Solid* lines connect the points. *Left*, total number (n) of motile filaments, *right*, speeds (mean and SD) from individual trials. *E*, NEM-Sk-S1 has no effect on (native-) Tpm3.1’s inhibition of 150 nM Myo1C gliding speed. Box and whiskers, median, quartiles, and range. Data are pooled from three days of trials with N = 2 preparations of Myo1C. Filament numbers, n, are labeled next to the plots including (n in parentheses) equally weighted data from trials where all of the filaments were nonmotile. Statistics, Kruskal–Wallis test.

**Figure S1:**
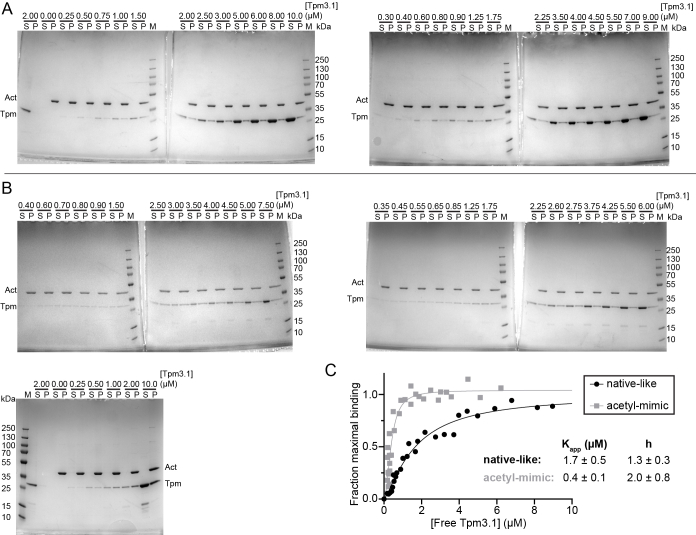


We measured the ability of Tpm3.1 with native amino (N)-terminal acetylation (native-) (33) to inhibit the *in vitro* activity of a Myo1C construct, consisting of the motor and 3-IQ containing lever arm domain (Myo1C-3xIQ) (34), in a gliding filament assay. The speed of actin gliding was reduced hyperbolically (IC_50_ = 0.7 ± 0.2 μM) as a function of increasing Tpm3.1 concentration (Fig. 1*A*). In contrast, myosin-II sliding velocity and ATPase increases hyperbolically or is unaffected as a function of Tpm concentration (2, 35, 36). Protein yields from the native-Tpm3.1 purifications were insufficient to perform experiments that require large amounts of Tpm. Therefore, we bacterially expressed and purified a high-yield Tpm3.1 construct with an N-terminal acetyl-mimicking modification (MAS-Tpm3.1) (37) and compared its actin affinity with native-Tpm3.1 by cosedimentation assay (Fig. S1). The affinity of MAS-Tpm3.1 (K_app_ = 0.4 ± 0.1 μM) for actin was tighter than the native-Tpm3.1 (K_app_ = 1.7 ± 0.5 μM) in our assay buffer, KMg25 (*p* < 0.0001, Extra sum-of-squares F-test). However, MAS-Tpm3.1 had a similar concentration-dependent effect on actin gliding as the native-like protein (IC_50_ = 0.7 ± 0.2 μM; Fig. 1*A*). Notably, neither Tpm proteins completely inhibited motility, with a minimum speed of 25 nm s^−1^ at saturating Tpm concentration, as observed previously (3, 5, 9). With no difference between the acetyl-mimic and the native-like Tpm3.1 constructs’ effects on gliding motility assays, we considered them interchangeable for practical use in regulating Myo1C activity. The absence of complete inhibition raised the question: does Tpm slow biochemical kinetic steps on the myosin ATPase pathway or does Tpm sterically exclude myosin binding to actin?

To address the potential that Tpm3.1 reduces the number of Myo1C heads bound to actin, we examined whether increasing the Myo1C density overcomes the Tpm3.1 inhibition of gliding speed. At the threshold of the minimum Myo1C concentrations required for motility, filament velocities were faster and with higher SDs than at higher Myo1C concentrations (Fig. 1*B*), which we attribute to diffusive motion interspersed between events of motile engagement between the filaments and motors (38). Persistent motility required 125 to 150 nM Myo1C for actin alone and 200 to 350 nM Myo1C for the native-Tpm3.1 condition (Fig. 1*B*), indicating that more Myo1C is required for minimal motility when actin is Tpm3.1-bound. This finding is consistent with previously published work employing Tpm1.6, Tpm1.7, and Tpm3.1 (3, 5, 9). Surprisingly, increasing the concentration of Myo1C applied to the motility surface by two orders of magnitude did not overcome Tpm3.1 inhibition (Fig. 1*B*). Steady-state assays revealed that Tpm3.1 strongly inhibits actin-activated Myo1C ATPase (0.001 ± 0.001 μM^−1^ s^−1^; *p* < 0.0001, F-test), whereas ATPase activity in the absence of Tpm3.1, 0.030 ± 0.003 μM^−1^ s^−1^, was consistent with previously reported rates (Fig. 1*C*) (34). Myo1C’s dependence on actin concentration was linear in the actin concentration range, thus maximal rates are not reported.

**Figure S2:**
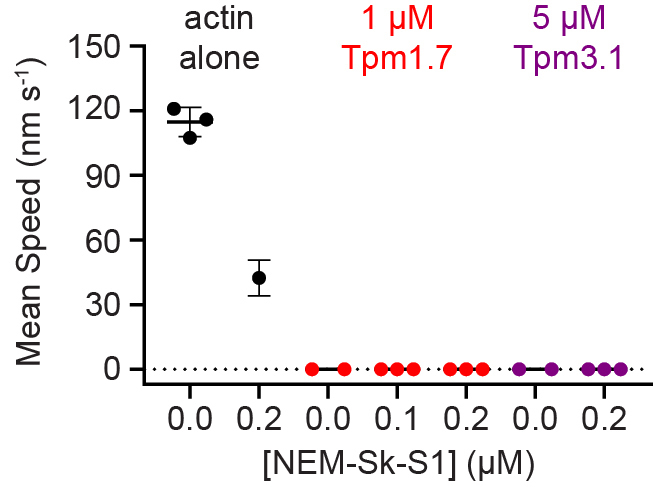


We attempted to cooperatively “activate” actin-Tpm3.1 by binding ATP-insensitive, N-ethylmaleimide (NEM)-treated, skeletal muscle myosin (Sk) heads (subfragment 1, S1) to actin. Strong binding of NEM-Sk-S1 to Tpm-actin has been shown to push tropomyosin into an “open” position that alleviates steric hinderance of some myosins (27, 28, 29). We initially varied the NEM-Sk-S1 concentration (0–1 μM) in actin-only (10 nM) motility assays to determine the effect of NEM-Sk-S1 on gliding speeds in the presence of 150 nM Myo1C. NEM-Sk-S1 (>0.2 μM) inhibited Myo1C actin-gliding in the absence of Tpm (Fig. 1*D*), which is an effect seen previously for actin alone in the presence of muscle myosin (29). We observed no actin filament-surface binding in the absence of Myo1C when 0.1 to 0.2 μM free NEM-Sk-S1 was included in the motility solution, thus the motility chambers were sufficiently blocked with casein to prevent nonspecific binding of NEM-Sk-S1, which would impart resisting load on motility. NEM-Sk-S1 (0.1–1.0 μM) was unable to rescue Myo1C motility in the presence of Tpm3.1 (Fig. 1*D*), whereas 0.2 μM NEM-Sk-S1 was shown to activate actin-Tpm filaments for muscle myosin gliding motility (29). Additional experiments were performed at 0.1 to 0.2 μM NEM-Sk-S1, which inhibited Myo1C gliding of actin alone (Figs. 1*E* and S2). Tpm3.1-actin gliding was not statistically changed by the presence of 0.1 to 0.2 μM NEM-Sk-S1 (Figs. 1*E* and S2). Together, these data suggest that Tpm3.1 inhibits Myo1C motility, even when the tropomyosin is moved to the “open” position on the actin filament (28). Thus, we next sought to determine whether Myo1C and Tpm3.1 can co-bind actin filaments.

**Figure 2:**
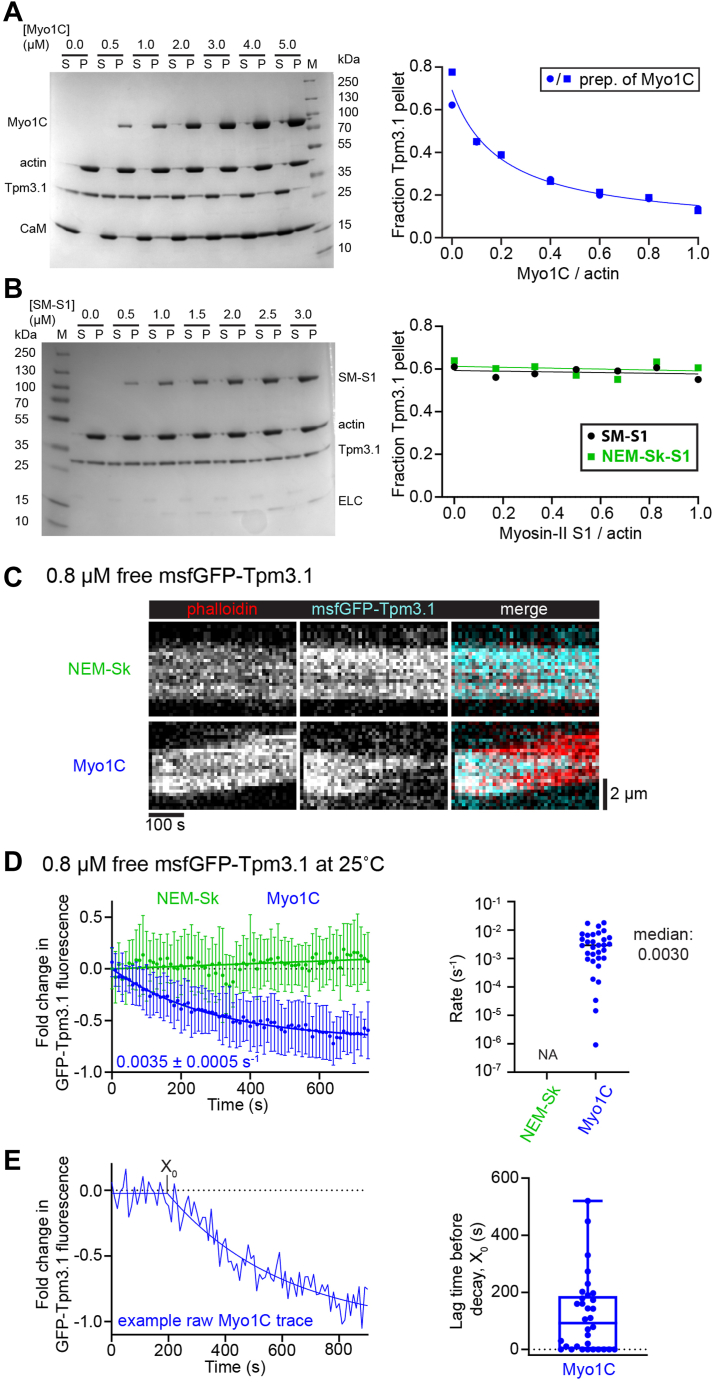
**Myo1C displaces Tpm3.1.***A* and *B*, cosedimentation assays with myosins (in rigor) and 2:1 actin-Tpm3.1. *Left*, example gel. *A*, Tpm3.1 sedimentation decreases with increasing Myo1C concentration (5 μM actin, 2.5 μM Tpm3.1). Data are from two trials and two preparations of Myo1C (*left* and Fig. S3A) Fit, dose-response inhibition model (IC_50_ = 1.0 ± 0.5 μM). Two additional trials in (Fig. S3*D*). *B*, class-II myosins binding in rigor have no effect on Tpm3.1 co-pelleting with actin (3 μM actin, 1.5 μM Tpm3.1). Data are from (*left*) smooth muscle myosin (SM)-S1 and (Fig. S3*B*) NEM-Sk-S1 (skeletal myosin). Linear fits have near-zero slopes. *A* and *B*, *right*, quantification of Tpm3.1 pelleting. (S) supernatant, (P) pellet, (M) molecular weight standards, (CaM) calmodulin light chains of Myo1C, (ELC) essential light chain MYL6. *C*, example kymographs from TIRF motility assays (see Experimental procedures) showing GFP-Tpm3.1, 0.8 μM in solution, associates with NEM-Sk myosin-bound filaments for many minutes but is gradually displaced from Myo1C-bound filaments undergoing motility (37 °C; see also Video S1). *D*, fluorescence transients, aligned at the start of decay (t = X_0_), from TIRF motility experiments (25 °C) of GFP-Tpm3.1 on individual filaments reveal that Tpm3.1 dissociates from filaments upon binding Myo1C, not NEM-Sk myosin. *Left*, data from multiple filaments were averaged together starting from (Myo1C) the beginning of decay or (NEM-Sk) start of the movie. Points, mean ± SD of averaged traces from (NEM-Sk) n = 22 and (Myo1C) n = 32 filaments. *Solid* lines, Myo1C data were fit to a single exponential (rate constant shown), while NEM-Sk have a linear fit (slope = 1.3 ± 0.6 x 10^−4^ s^−1^). *Right*, rates from exponential fits from individual filaments. Data are pooled from three trials with N = 3 preparations of Myo1C. *E*, initial lag times (X_0_) before decay of GFP-Tpm fluorescence from Myo1C-filaments. *Left*, example of fluorescence decay from an individual filament. *Right*, X_0_ from data in *D*.

**Figure S3:**
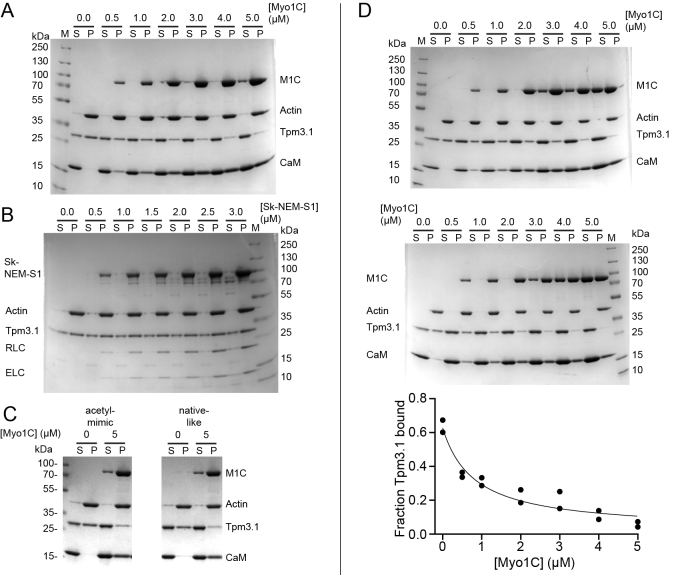


We performed cosedimentation assays to determine if nucleotide-free Myo1C could bind Tpm3.1-actin filaments (2.5 μM Tpm and 5 μM actin). Strikingly, increasing concentrations of Myo1C hyperbolically decreased both MAS- and native- Tpm3.1 co-pelleting with actin filaments (Figs. 2*A* and S3, *A*, *C*, and *D*). In contrast, smooth muscle myosin (SM)-S1 and NEM-Sk-S1 rigor binding had no effect on Tpm3.1 co-pelleting (Figs. 2*B* and S3*B*), consistent with the conventional closed-open state model for tropomyosin regulation (28).

We next determined whether Tpm3.1 displacement occurs in motility assays. To visualize Tpm during actin gliding, we generated Tpm fusion constructs with a monomeric, photostable variant of superfolder (msf) EGFP (39) using an established acetyl-mimicking amino (N)-terminal linker (GSMAS) (7, 25) (see Experimental procedures). We performed motility assays as above, but with 0.8 μM GFP-Tpm3.1 free in solution and used total internal reflection fluorescence (TIRF) microscopy to image filament-bound GFP-Tpm3.1 near the coverslip surface. GFP-Tpm3.1-actin filaments attached to the surface by NEM-Sk myosin retained fluorescence for the duration of our experiments (700 s; Fig. 2*C* and Video S1). For filaments, undergoing motility driven by Myo1C, GFP-Tpm3.1 was initially bound to the filaments but was partially displaced over time (Fig. 2*C* and Video S1). We measured the kinetics of GFP-Tpm3.1 displacement at 25 °C to achieve photostability of the GFP, which resulted in slowed actin gliding. GFP-Tpm3.1 dissociated from actin in the presence of Myo1C at a rate of 3.5 ± 0.5 × 10^−3^ s^−1^ (Fig. 2*D*), following a refractory period (Fig. 2*E*). These data indicate that the Myo1C-coated surface binds to Tpm3.1-coated filaments and weakens the affinity of Tpm3.1, leading to displacement of Tpm3.1 from actin in gliding motility assays.

**Figure 3:**
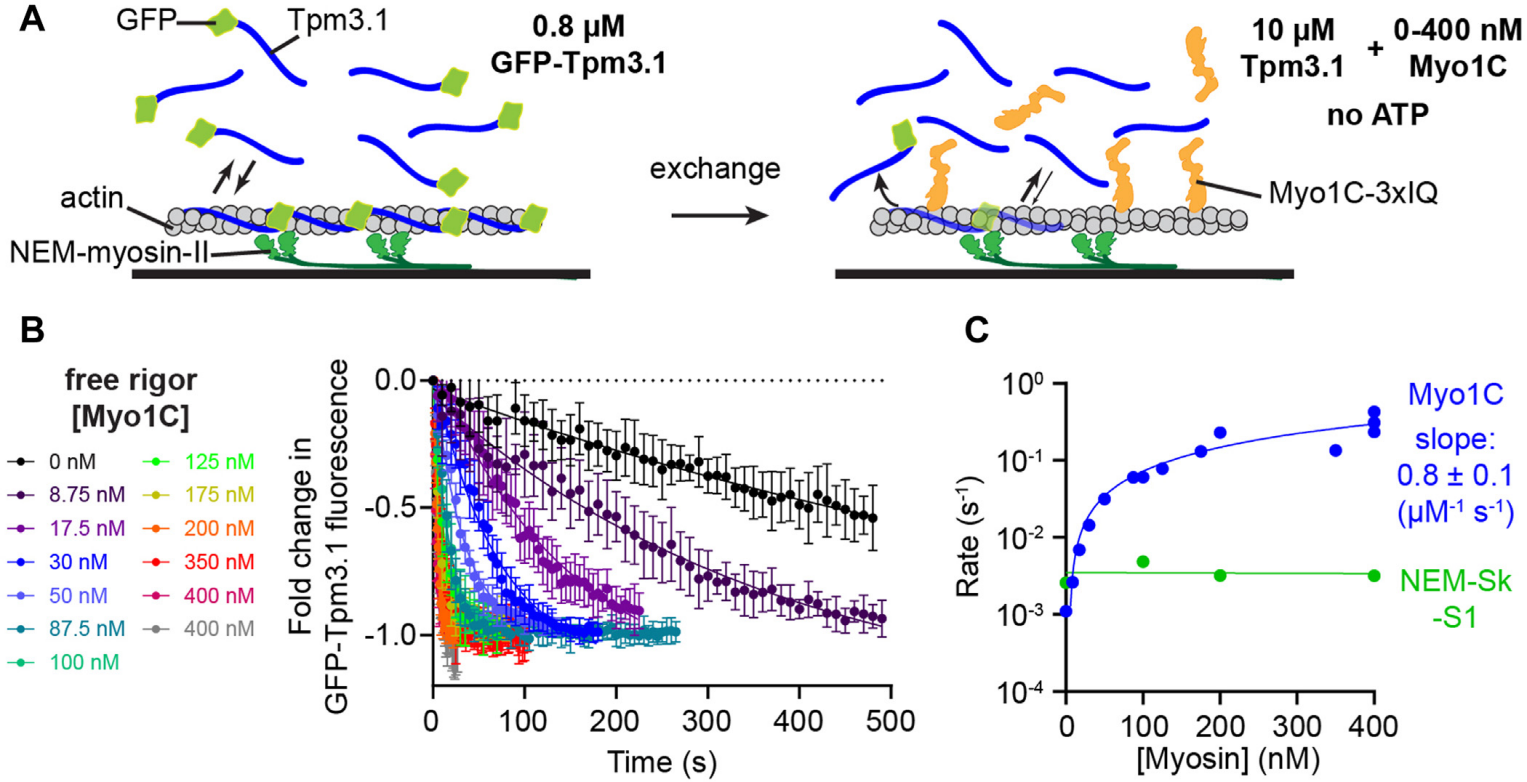
**Kinetics of GFP-Tpm3.1 dissociation.***A*, TIRF GFP-Tpm3.1 displacement assays (25 °C) where reaction solutions were exchanged from 0.8 μM GFP-Tpm3.1 to 10 μM unlabeled Tpm3.1 with variable concentrations of free Myo1C (rigor) or NEM-Sk-S1 (*C*; *green*). *B*, averages of filaments (±SD) from each Myo1C concentration (color-coded to the left) fit to exponentials. *C*, apparent rate constants (log scale) with linear fits giving the second-order rate constant. Data are pooled from three days of trials with n = 10 filaments per condition, N = 2 preparations of Myo1C.

We determined the rate of Myo1C-induced GFP-Tpm3.1 dissociation from actin by mixing immobilized GFP-Tpm3.1-actin filaments with 10 μM unlabeled Tpm3.1 and increasing concentrations of free Myo1C in the absence of ATP. The rate of GFP-Tpm3.1 exchange increased linearly with increasing Myo1C (0.8 ± 0.1 μM^−1^ s^−1^; Fig. 3), with rates up to 0.4 s^−1^ achieved at 400 nM Myo1C, which was ∼400-fold faster than the basal rate of exchange (1.1 ± 0.7 × 10^−3^ s^−1^). Replacing Myo1C with increasing concentrations of NEM-Sk-S1 did not increase the rate of GFP-Tpm3.1 dissociation (Fig. 3*C*). Importantly, these data show that Myo1C binding increases the rate of GFP-Tpm3.1 displacement. Interestingly, the rate of GFP-Tpm3.1 dissociation in the absence of free protein (9 ± 1 × 10^−3^ s^−1^) is approximately tenfold faster than in the presence of 10 μM free Tpm3.1 (1 ± 1 × 10^−3^; Fig. S4, *A* and *B*). The likely explanation of these data is that the unlabeled Tpm slowed the rate of GFP-Tpm3.1 dissociation because of its cooperative binding (33, 40, 41, 42), accordingly Tpm3.1 requires a critical concentration of free subunits to decorate actin, and subunits at the ends of the Tpm3.1 polymers are in rapid exchange with the solution. Together, the data show that Myo1C-actin strong-binding accelerated GFP-Tpm3.1 dissociation.

**Figure 4:**
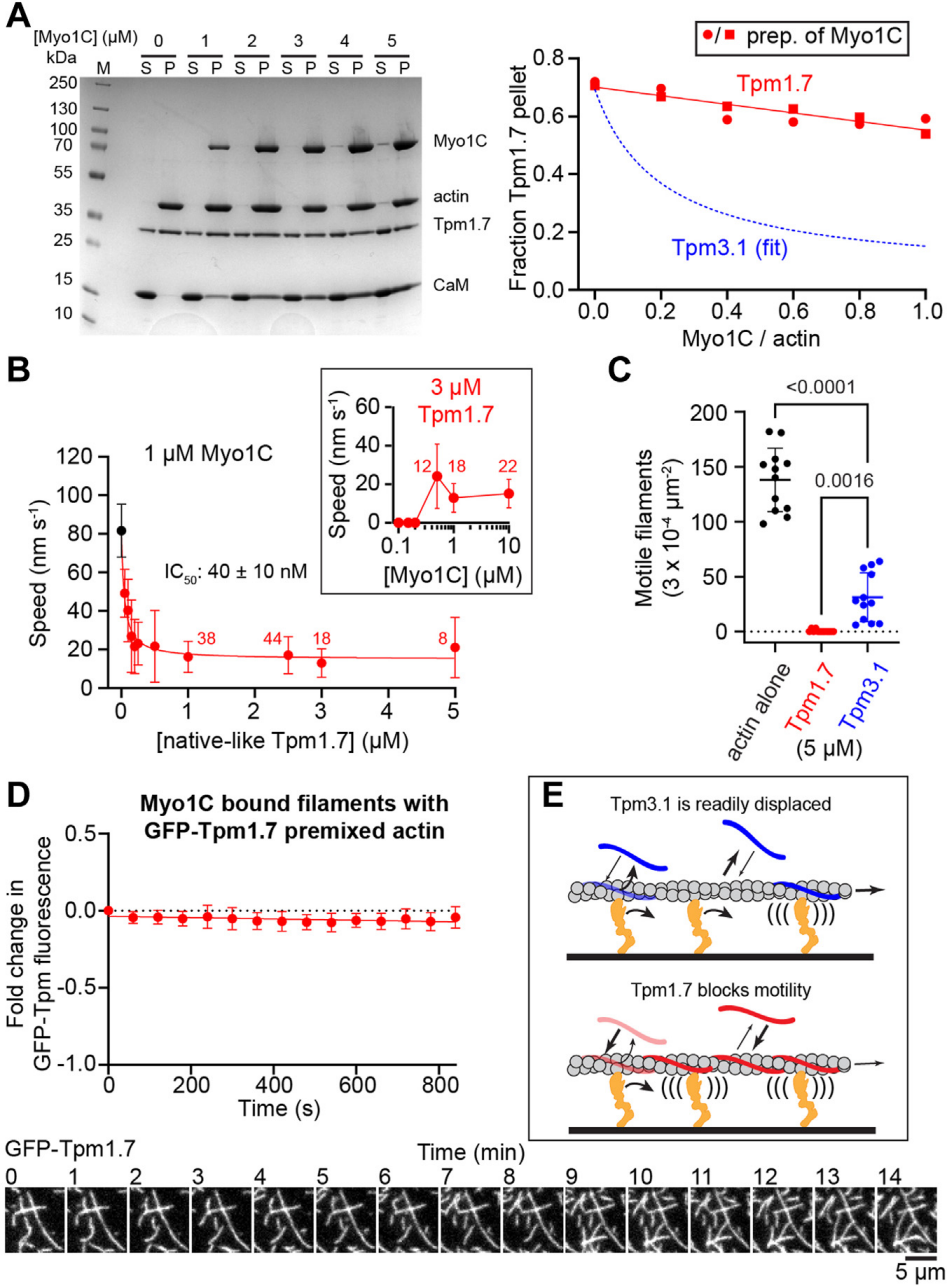
**Tpm1.7 binds acto-Myo1C and inhibits gliding more than Tpm3.1.***A*, cosedimentation assays with Myo1C (in rigor) and 2:1 actin-Tpm1.7 show that displacement of Tpm1.7 is 75% less than Tpm3.1 (Fig. 2*A*). *Left*, example gel. *Right*, quantification of Tpm1.7 pelleting. Tpm3.1 fit (*blue*) taken from Figure 2*A*. (S) supernatant, (P) pellet, (M) molecular weight standards, (CaM) calmodulin. Data are from two trials and N = 2 preparations of Myo1C (*left* and Fig. S4*C*). Slope of linear fit: −3 ± 1 x 10^−2^ μM^−1^. An additional trial in Supplemental (Fig. S4*D*). *B*, inhibition of Myo1C gliding motility speed by Tpm1.7. Inset, Tpm1.7 inhibits speed over a range of Myo1C concentrations. Points, mean ± SD of each trial, n = 50 filaments, unless labeled. *C*, Tpm3.1 and Tpm1.7 reduce the number of filaments glided by Myo1C, with Tpm1.7 significantly inhibiting motility more than Tpm3.1. Median speeds: actin alone, 97 nm s^−1^; Tpm1.7, 15 nm s^−1^; and Tpm3.1, 27 nm s^−1^. Data are from N = 3 preparations of Myo1C, with conditions performed side-by-side. Statistics, Brown-Forsythe ANOVA. *D*, GFP-Tpm1.7 stays bound when pre-mixed 1:4 with actin in Myo1C TIRF gliding assays (final concentrations: 2.5 nM GFP-Tpm1.7, 10 nM actin, 25 °C). *Top*, mean ± SD pooled from 3 trials, n = 15 filaments, and N = 3 preparations of Myo1C. *Bottom*, example image series showing phalloidin-stabilized filaments (unlabeled) decorated with GFP-Tpm1.7. *E*, model of Myo1C motility and displacement of Tpm3.1 *versus* Tpm1.7.

To determine whether Myo1C-induced displacement of Tpm depends on the Tpm isoform, we performed cosedimentation experiments with (native-) Tpm1.7 in the presence of nucleotide-free Myo1C as above. Tpm1.7 binding to actin was only marginally reduced by strong-binding of Myo1C, in contrast to the almost complete displacement of Tpm3.1 (Figs. 4*A* and S4, *C* and *D*). Tpm1.7 inhibited the actin gliding speed to 15 ± 1 nm s^−1^ in motility assays with an IC_50_ = 40 ± 10 nM (Fig. 4*B*), ∼20-fold lower than Tpm3.1 (Fig. 1*A*), consistent with Tpm1.7 having a higher affinity for actin (7). As with Tpm3.1-actin, increasing the Myo1C concentration did not increase the speed of Tpm1.7-actin gliding (Fig. 4*B*). At 150 nM Myo1C (as employed in Fig. 1, *D* and *E*), 0.1 to 0.2 μM free NEM-Sk-S1 had no effect on the inhibition of Myo1C motility by Tpm1.7 (Fig. S2), indicating that myosin-II binding did not “activate” Tpm-actin for Myo1C with either isoform. In Myo1C gliding motility assays, there were substantially fewer motile actin filaments in the presence of Tpm1.7 than in the absence of tropomyosin or presence of Tpm3.1 (Fig. 4*C*). Unlike GFP-3.1, GFP-Tpm1.7 was not displaced from Myo1C-bound filaments in gliding assays (Figs. 4*D* and S4, *E*–*G*). Thus, our results show that Myo1C cannot displace all Tpm isoforms equally and the ability to do so alters the inhibition of motility by Tpm.

## Discussion

Here, we find that Myo1C activity is more strongly inhibited by Tpm1.7 than by Tpm3.1, with an important difference between these isoforms being that Tpm3.1 is readily displaced by Myo1C while Tpm1.7 is not (Fig. 4*E*). Additionally, our results shed new light on the biochemical mechanism of inhibition of Myo1C by nonmuscle Tpms. Overall, Myo1C’s isoform-dependent displacement of and regulation by Tpm isoforms offer intriguing insights that are likely to reveal key underpinnings of the intracellular mechanochemical sorting of Tpm isoforms and unconventional myosins.

Our results support a partial steric blocking mechanism of inhibition, where achieving the “open” conformation of Tpm is insufficient to achieve the full binding and activation of Myo1C on actin. The near abolition of actin-activated ATPase activity in the presence of excess Tpm3.1 (Fig. 1*C*) suggests that Tpm blocks phosphate release, the rate-limiting step for acto-Myo1C ATPase (34). Residual ATPase and motility activities are likely due to the displacement of Tpm by Myo1C (Figs. 1 and 4). Minimal gliding motility requires higher Myo1C concentrations in the presence of Tpm than for actin alone (Fig. 1*B*), which points to a weakened acto-Myo1C affinity. Taken together, these observations suggest it is likely that phosphate release is blocked by preventing the transition from weak to strong actin-binding. Moreover, we show that myosin-II binding fails to “activate” Tpm-actin for Myo1C, in that it fails to restore actin binding and motility speed to actin-only levels (Fig. 1, *D* and *E*). This result indicates that the open state created by myosin-II is not fully open to Myo1C, and it is likely that only Tpm-free actin allows Myo1C’s complete activation. The unavailability of the open state would ensure the filaments activated by other myosins would still not be a substrate for Myo1C, which could explain why myosin-I is excluded from myosin-II- and Tpm-containing stress fibers (12).

A previous study concluded that Tpms’ inhibition of Myo1C gliding speed is due to changes in the tension sensitivity of the motor domain (9). However, with our new understanding of the relationship between strong-binding of myosin and tropomyosin dissociation, we now conclude that the rate of motility is more likely limited by the rate of Tpm dissociation.

Despite the low apparent affinity of Myo1C for Tpm-actin, we see binding of Tpm-actin to Myo1C in motility assays. This binding is unlikely due to Loop-2 interaction with actin, as this loop does not include charged residues found in other myosins (*e.g.*, myosin-II and myosin-V) important for binding in the pre-force states. Rather, we propose that binding of the lower 50 kDa region of Myo1C is responsible for this initial binding, as recently reported for myosin-V (43). For the power stroke to occur and phosphate to be released, the actin-binding cleft must close, which involves the movement of loop-4 and the cardiomyopathy loop. Recent computational work modeling cardiac myosin’s interaction with thin filaments suggests the closing of the actin-binding cleft, and charge repulsion between residues of loop-4 and Tpm result in the translocation of Tpm across actin to achieve the open state (30). The charge of the Tpm-interacting end of loop-4 in Myo1C (Asp^322^-Glu^323^-Asp^324^) is substantially different than the corresponding residues of β-cardiac myosin (Gln^368^-Arg^369^-Glu^370^) (44). Tpms all share conserved charged residues periodically located along their lengths that facilitate actin and myosin binding (29, 45, 46). Notably, the Tpm residues that interact with myosin are predominantly negative (29). The resulting clash between Myo1C’s loop-4 and Tpm is likely to prevent Myo1C’s release of phosphate and swing of the lever, consistent with the inhibition we observed (Fig. 1*C*). Our unexpected finding that high densities of Myo1C can displace Tpm reveals that slow motility is likely occurring under a mixed population of motors, some of them occluded by Tpm and some uninhibited because Tpm has been displaced. In support of this notion, Myo1C displaces Tpm1.7 weakly compared to Tpm3.1, while Tpm1.7 inhibits Myo1C more strongly, thus displacement correlates to the relative frequency of slow motility events.

Based on these and previous experiments, inhibition by nonmuscle Tpms appears to be a key factor in regulating myosin-I localization and function (3, 5, 11, 12). Strikingly, Tpms have opposing effects on nonmuscle myosin-II paralogs, as their ATPase rates and motility are increased by Tpms, especially by short isoforms Tpm3.1, Tpm3.2, and Tpm4.2 (2, 7, 9). Additionally, myosin-V motility is also enhanced by Tpms in some contexts (6, 8, 47). In fungal systems where myosin–Tpm interactions have been studied additionally, Tpms also inhibit myosin-I while enhancing myosins -II and -V (4, 10, 15, 48, 49, 50, 51, 52), indicating that Tpms coevolved with myosins to segregate their functions in this class-specific manner.

The question remains as to whether locally high concentrations of Myo1C in the cell exclude Tpm localization. For example, intestinal epithelial cell microvilli contain high concentrations of Myo1A and exclude Tpm, despite Tpm’s concentration in the adjacent terminal web of actin (11, 53). Myo1C is enriched at regions of the plasma membrane and at several other intracellular lipid membranes, where it may encounter Tpm-decorated filaments, but cellular Myo1C activities are correlated mainly with Tpm-free Arp2/3 complex-nucleated branched actin networks (54). Tpm1.7 and Tpm3.1 are found in stress fibers and the cleavage furrow, Tpm1.7 localizes to filopodia, and Tpm3.1 localizes to many other actin structures including the cell cortex (1). Future studies quantitatively measuring the local concentrations and position of Myo1C compared to Tpm1.7 and Tpm3.1 in cells will further elucidate the cellular relevance of our *in vitro* work. Based on our findings that Myo1C displaces Tpm *in vitro*, we speculate that myosin-I may play an early upstream role in biasing structures, such as the leading edge of lamellipodia, against binding Tpm. Through its tail-based recruitment to membranes in parallel with actin assembly, myosin-I is in a unique position to bind actin filaments upstream of other filament side-binding proteins, including Tpm, and thus myosin-I may bias which other components can competitively bind. Intriguingly, micro-injection of exogenous Tpm1.7, but not Tpm3.1, into live cells inhibits the perinuclear accumulation of organelles which is mediated by Myo1C (32). Our results suggest this differential effect could be due to the inability of Tpm3.1 to displace Myo1C, unlike Tpm1.7 which effectively blocks Myo1C motility. The mechanisms by which Tpm isoforms are differentially recruited remain mysterious. While it has been hypothesized that formins may directly recruit Tpm isoforms, reconstitution studies instead suggest that Tpm is excluded through internetwork competition and combinatorial synergy between multiple actin-binding proteins (10, 55, 56, 57, 58). Supporting the latter model, Tpm isoform expression levels in mammalian cells, not the formins mDia1 and mDia3, were found to determine the concentrations of each isoform bound to actin filaments (59). Further studies should define the rules for self-sorting among various myosins, other actin binding proteins, and Tpm isoforms.

## Experimental procedures

### Proteins

Myo1C-3xIQ-AviTag-FLAG, containing the amino(N)-terminus through all three IQ domains (Q9WTI7-2, residues 1–767) immediately followed by the sequence GGLNDIFEAQKIEWHEAADYKDDDDK that includes a BirA biotinylation site (AviTag; GLNDIFEAQKIEWHE) and a FLAG epitope tag (DYKDDDDK) for purification, was expressed using the Sf9-baculovirus system and purified by FLAG-affinity and ion exchange as previously described (34, 60, 61). Recombinant versions of human Tpm3.1 and Tpm1.7 including the native-like expressed in Expi293 cells or the acetyl-mimic (with N-terminal Met-Ala-Ser extension) expressed in *Escherichia coli* were purified using established constructs and methods (33, 62). Briefly, native-like Tpm3.1 and Tpm1.7 were expressed fused to tandem carboxy(C)-terminal intein and chitin-binding protein tags such that intein-based self-digestion following chitin affinity purification produced endogenously N-terminal acetylated Tpm3.1 and Tpm1.7 with no additional residues (33).

Constructs in the pET3a backbone were synthesized to express poly-His (MGHHHHHHGSGSG)-monomeric superfolder (msf)EGFP fused N-terminally to Tpm3.1 and Tpm1.7 through a GSMAS acetyl mimicking linker established by Peter Gunning et al. (7, 25, 63). The msfEGFP was employed to enhance the expression and stability of the fusion constructs while retaining monomeric and photostable properties and contained the original superfolder mutations, except for the photostabilizing Y145 and the monomerizing A206K (39), which we refer to simply as GFP. GFP-Tpms were purified by Dounce homogenization of *E. coli* pellets into NEBExpress lysis buffer (New England Biolabs) with added 0.5 mM DTT (dithiothreitol), 1 mM PMSF (phenylmethylsulfonyl fluoride), 10 μg mL^−1^ leupeptin, and 10 μg mL^−1^ aprotinin, centrifugation at 48,000*g* for 40 min at 4 °C, loading of clarified lysates over a 20 ml packed Nickel NTA Agarose resin bed (Goldbio), washing with three cv of buffer A (25 mM tris, pH 8, 10 mM imidazole pH 7.5, 300 mM NaCl, 1 mM EGTA [ethylene glycol tetraacetic acid], and 1 mM sodium azide), elusion in buffer A with 250 mM imidazole, and dialysis of peak fractions into GS buffer (50% glycerol, 50 mM tris, pH 8, 50 mM KCl, 1 mM MgCl2, 1 mM EGTA, and 2 mM DTT).

Rabbit skeletal muscle actin was purified as previously described (64) and stabilized with phalloidin in all experiments (fluorescently labeled or unlabeled).

### Cosedimentation assays

Actin, Myo1C, 10 μM calmodulin (CaM), and Tpm were mixed, as indicated, in KMg25 (60 mM 3-(N-morpholino)propanesulfonic acid, pH 7.0, 1 mM MgCl_2_, 1 mM EGTA, and 20 mM DTT), incubated for 30 min at room temperature, and then centrifuged at 200,000*g* for 30 min at 25 °C. Supernatant (S) and pellet (P) fractions were suspended in equal total volumes of SDS sample buffer and loaded equally for SDS-PAGE. For Tpm-actin binding assays (without Myo1C), the actin concentration was 7 μM and Tpm3.1 concentration was varied, as indicated. For assays of Tpm displacement by rigor Myo1C, actin and Tpm were held constant (5 μM actin and 2.5 μM Tpm, unless stated otherwise), while Myo1C was added at the indicated concentrations (0–5 μM). Apyrase at 1U/ml was added to ensure Myo1C was nucleotide-free. The Coomassie-stained gels were analyzed by gel densitometry using ImageJ. Fractions bound and free were calculated based on the ratio of the fractions and the total concentration. The data were also corrected to account for variability in the amount of actin in the P fraction ((P ∗ actin ratio)/(S + P ∗ actin ratio)). Points (Fig. S1*C*) were fit to specific binding with Hill slope equation, Y = B_max_ ∗ X^h^/(K_d_^h^ + X^h^).

### Motility assays

Flow chambers were assembled with nitrocellulose-coated coverslips as previously described (65). Biotinylated Myo1C was attached to the coverslip by the sequential addition of 0.1 mg mL^−1^ neutravidin, 2 mg mL^−1^ casein, and then variable concentrations of Myo1C in buffer M (10 mM imidazole pH 7.5, 300 mM NaCl, 5 mM MgCl_2_, 1 mM EGTA, 2 mM DTT, and 4 μM CaM). Subsequently, motility reactions were initiated by the addition of 10 nM actin filaments in KMg25 supplemented with motility components (2 mM MgATP, 0.2 mg mL^−1^ glucose oxidase, 40 μg mL^−1^ catalase, and 5 mg mL^−1^ glucose). Before imaging, motility reactions were equilibrated to 37 °C by the heated objective for 2 min. For tropomyosin decoration, actin filaments were both pre-mixed 1:1 with Tpm3.1 and excess Tpm3.1 was added in the motility reaction buffer, except for experiments where the concentration of free Tpm3.1 was varied without pre-mixing. Velocity analysis was performed using the MTrackJ plugin in ImageJ. In Figure 1*E*, individual trials with zero and non-zero speeds within each condition were weighted equally by including proportional numbers of zeros.

Motility assays employing TIRF microscopy to track GFP-Tpm fluorescence were performed at 37 °C (the standard condition) or at 25 °C to measure GFP-Tpm dissociation kinetics, as indicated. Actin filament attachment was either through Myo1C gliding, as above, or by the sequential addition of 0.1 mg mL^−1^ NEM-treated skeletal muscle myosin, 2 mg mL^−1^ casein, and then reaction mixes, which were identical to above, except the inclusion of GFP/unlabeled-Tpm (as indicated) and 0.2 mg mL^−1^ casein. At 37 °C, the casein concentrations were increased fivefold to promote GFP-Tpm binding specificity. For Myo1C conditions, 1.7 μM Myo1C was applied to the motility surface through neutravidin attachment. Excess CaM (30 μM) was added to all motility reaction mixtures. During exchange, additional actin was not included.

### Steady-state ATPase measurements

The NADH-coupled assay was used to measure steady-state Myo1C ATPase activity at 37 °C in KMg25 (with 1 mM DTT) as previously described (34, 66). The final concentrations after mixing were as follows: 100 nM Myo1C, 10 μM free CaM, and 0 to 100 μM actin, employing three different preparations of Myo1C. To ensure effective competition with Myo1C, Tpm3.1 was added in excess to the reaction in addition to being preincubated with actin. Therefore, these conditions ranged from 15 to 60 μM Tpm3.1, increasing with actin concentration. A Photon Technology International fluorometer was used to collect fluorescence measurements.

## Graphing and statistics

Graphs and statistics were generated using GraphPad Prism 10.2.0 (GraphPad Software, Inc).

## Data availability

Data to be shared upon request to E. Michael Ostap (ostap@pennmedicine.upenn.edu).

## Supporting information

This article contains supporting information (37).

## Conflicts of interest

The authors declare that they have no conflicts of interest with the contents of this article.

## Figures and Tables

**Figure S4:**