# Supplementary material for: Myosin-1C differentially displaces tropomyosin isoforms altering their inhibition of motility
Source: J Biol Chem. 2024 Jul 4;300(8):107539. doi: 10.1016/j.jbc.2024.107539 (PMC11338116; doi:10.1016/j.jbc.2024.107539)
Supplement: Supporting Information [file mmc1.docx]

**Supporting Information**

**Figure S1. Cosedimentation assays of Tpm3.1 binding to actin.** (A) Gels of native-like Tpm3.1 and 7 µM actin. (B) Gels of acetyl-mimic Tpm3.1 and 7 µM actin. (C) Densitometry analysis of A-B fit to Hill curves (solid lines). S, supernatant, P, pellet, M, molecular wight standards, Act, actin, Tpm, Tpm3.1. Acetyl mimic Tpms have been shown to readily polymerize on their own under low ionic strength (1), which we also observed as excess pelleting under high concentrations (10 µM; B). Therefore, the acetyl-mimic was employed within the regime of the initial plateau. The higher apparent affinity may be accounted for by the acetyl-mimic’s propensity to self-polymerize in 25 mM KCl, since polymerization enhances actin binding. Together, these data suggest that the acetyl-mimic may perform better in regulating actin gliding motility by requiring less free Tpm to saturate actin.

**Figure S2. Effect of NEM-Sk-S1 on Myo1C motility with Tpm1.7 or Tpm3.1.** Gliding-filament motility assays performed with 150 nM Myo1C (at the threshold of minimal motility with actin alone). Native-Tpm was used for both isoforms. Points, mean speeds from individual trials with n = 20 filaments each (except when motility is abolished), horizontal lines, means of combined trials, error, SD. Data are from N = 1 preparation of Myo1C. One-way ANOVA: Tpm1.7 and Tpm3.1 exhibited no motility while actin alone had a speed of 115 ± 7 nm s^-1^ (p < 0.0001), whereas 0.1-0.2 µM NEM-Sk-S1 had no effect on either Tpm condition (p > 0.9999).

**Figure S3. Cosedimentation assays of myosins and actin-Tpm3.1.** (A) Gel of the repeat experiment included in Figure 2A (5 µM actin, 2.5 µM Tpm3.1). (B) Gel of experiment using NEM-treated skeletal muscle myosin S1 (Sk-NEM-S1) included in Figure 2B (3 µM actin, 1.5 µM Tpm3.1). (C) Cosedimentation assays showing that both acetyl-mimic and native-like Tpm3.1 are displaced by 5 µM Myo1C (5 µM actin, 2.5 µM Tpm3.1). (D) Cosedimentation experiments showing displacement of Tpm3.1 by Myo1C with 2.5 µM actin and 2.5 µM Tpm3.1. Above, gels, below, densitometry analysis of above. Solid line, dose-response inhibition model (IC_50_ = 0.4 ± 0.3 µM). S, supernatant, P, pellet, M, molecular wight standards, M1C, Myo1C, CaM, calmodulin, RLC, regulatory light chain, ELC, essential light chain.

**Figure S4. Binding of Tpm3.1 and Tpm1.7 to Myo1C- or NEM-Sk-bound actin filaments.** TIRF gliding assays (25˚C with ATP) where reaction solutions were exchanged from 0.8 µM GFP-Tpm3.1 to (A) no free Tpm or (B) 10 µM unlabeled Tpm3.1. (A-B) Filaments were bound to the TIRF surface with either NEM-Sk-myosin (green) or Myo1C (blue). Left, averages of multiple filaments (± SD) fit to single exponentials. Right rates from exponential fits of individual filaments. (A) NEM-Sk: n = 15 filaments, Myo1C: n = 18, N = 1 preparation of Myo1C. (B) NEM-Sk: n = 35 filaments, Myo1C: n = 32, N = 3 preparations of Myo1C. (C) Gel of the repeat Tpm1.7 cosedimentation experiment included in Figure 4A (5 µM actin, 2.5 µM Tpm1.7). (D) Cosedimentation experiment varying Myo1C concentration with 3 µM actin and 3 µM Tpm1.7. Left, Gel, right, densitometry. (Line) Linear fit, slope = -2 ± 2 x 10^-2^ µM^-1^. (E) Change in fluorescence of GFP-Tpm1.7 on filaments in TIRF Myo1C gliding assays with 0.8 µM GFP-Tpm1.7 free in solution. Points, mean ± SD of n = 20 filaments, N = 2 preparations of Myo1C. Tpm3.1 fit taken from Figure 2D. (F) GFP-Tpm1.7 fluorescence on Myo1C-bound filaments when free Tpm is removed by exchange. Points, mean ± SD of n = 20 filaments, N = 1 preparations of Myo1C. Tpm3.1 fit taken from A. (G) Example series from data in F showing GFP-Tpm1.7 on a filament after exchange. Experiments were performed at 25˚C.

**Video 1. Actin gliding motility at 37˚C with 0.8 µM free GFP-Tpm3.1.** NEM-Sk myosin was used as a control of GFP-Tpm3.1 binding to static filaments. Myo1C bound filaments are undergoing motility while displacing the GFP-Tpm3.1.

**SI References**

1. Monteiro, P. B., Lataro, R. C., Ferro, J. A., and Reinach Fde, C. (1994) Functional alpha-tropomyosin produced in Escherichia coli. A dipeptide extension can substitute the amino-terminal acetyl group. *J Biol Chem* **269**, 10461-10466
